# The resolution of inflammation through omega-3 fatty acids in atherosclerosis, intimal hyperplasia, and vascular calcification

**DOI:** 10.1007/s00281-019-00767-y

**Published:** 2019-11-06

**Authors:** Miguel Carracedo, Gonzalo Artiach, Hildur Arnardottir, Magnus Bäck

**Affiliations:** 1 Translational Cardiology, Department of Medicine, Neo, Karolinska Institutet, Stockholm, Sweden; 2 Theme Heart and Vessels, Division of Coronary and Valvular Heart Disease, Karolinska University Hospital, M85, 14186 Stockholm, Sweden

**Keywords:** Inflammation, Lipoxygenase, Resolvins, Vascular injury

## Abstract

Omega-3 fatty acids serve as the substrate for the formation of a group of lipid mediators that mediate the resolution of inflammation. The cardiovascular inflammatory response in atherosclerosis and vascular injury is characterized by a failure in the resolution of inflammation, resulting in a chronic inflammatory response. The proresolving lipid mediator resolvin E1 (RvE1) is formed by enzymatic conversion of the omega-3 fatty acid eicosapentaenoic acid (EPA), and signals resolution of inflammation through its receptor ChemR23. Importantly, the resolution of cardiovascular inflammation is an active, multifactorial process that involves modulation of the immune response, direct actions on the vascular wall, as well as close interactions between macrophages and vascular smooth muscle cells. Promoting anti-atherogenic signalling through the stimulation of endogenous resolution of inflammation pathways may provide a novel therapeutic strategy in cardiovascular prevention.

## Introduction

The inflammatory response in atherosclerosis is characterized both by a continuous immune activation [1] and a failure in the resolution of inflammation [2]. Several cardiovascular risk factors, including hypercholesterolemia, diabetes, smoking, and obesity, are associated with increased systemic chronic inflammation. Lowering low-density lipoprotein (LDL) cholesterol decreases inflammatory markers such as CRP, emphasizing the close interplay between lipids and inflammation [3]. However, the substantial residual risk despite the achievement of LDL cholesterol treatment goals and the observations that inflammatory markers predict cardiovascular risk independently of LDL cholesterol imply a treatment benefit of specifically targeting inflammation in atherosclerosis [4]. This was recently reinforced by the clinical benefit of targeting interleukin (IL) 1β for secondary prevention in high-risk patients [5]. Some concerns have however been raised as to the risk of immunosuppression and, consequently, increased incidence of infections in patients on anti-inflammatory treatments, both in in general and specifically in the context of cardiovascular prevention [5].

As an alternative to inhibiting proinflammatory signalling, cardiovascular inflammation could potentially be disrupted by actively turning on/promoting a functional and effective resolution of inflammation [2]. In that context, recent evidence indicate a deficiency of proresolving mediators in atherosclerotic lesions with an imbalance towards increased proinflammatory signalling [2]. One such example is bioactive lipid mediators derived from lipoxygenation of polyunsaturated fatty acids (PUFAs). The 5-lipoxygenase (5-LO) metabolism of arachidonic acid into proinflammatory leukotrienes (LT), will transduce several proatherosclerotic signals through their specific receptors [6]. On the other hand, double lipoxygenation of arachidonic acid via sequential actions of two lipoxygenase enzymes (i.e., 5-LO and either 12- LO or 15-LO) results in formation of different class of lipid mediators termed lipoxins (LX) that protect against atherosclerosis [7]. Indeed, the levels of leukotrienes increase whereas lipoxins decrease in both experimental and clinical atherosclerosis [8–11].

### Omega-3 PUFA-derived proresolving lipid mediators

**Fig. 1:**
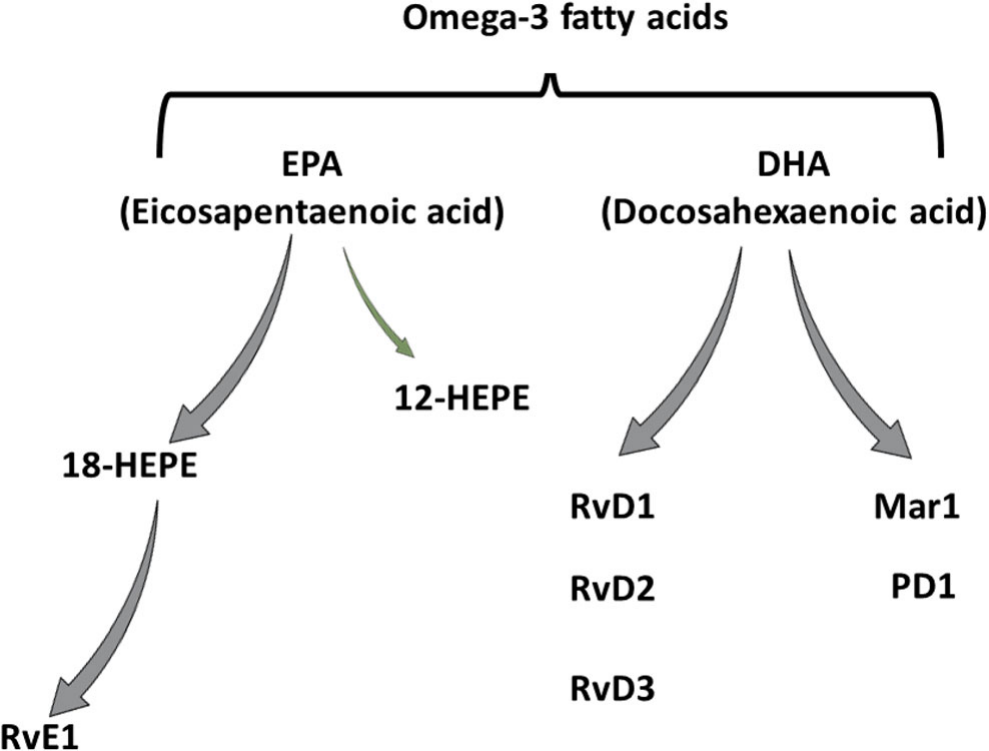
Specialized proresolving mediators (SPM) derived from omega-3 polyunsaturated fatty acids. Abbreviations: Rv, resolvin; MaR1, maresin 1; PD1, protectin 1

Another group of proresolving lipid mediators that have received recent attention in atherosclerosis is derived from LO metabolism of omega-3 PUFAs [12]. Specifically, omega-3 PUFAs serve as the substrate for the formation of D-series resolvins (RvD), maresins(MaR), and protectins (PD) from docosahexaenoic acid (DHA) and E-series resolvins (RvE) from eicosapentaenoic acid (EPA) (Fig. 1), which together with the lipoxins collectively have been devised specialized proresolving mediators (SPMs) [12]. SPMs exert their actions through specific G-protein-coupled receptors (GPCR), namely, ChemR23 and BLT1 for RvE1, ALX/FPR2 and GPR32 for RvD1 and RvD3, and GPR18 for RvD2 [2].

The Rv to LT ratio has emerged as a measure of non-resolving cardiovascular inflammation [13, 14]. This is important since it not only lends further support to the concept that atherosclerosis is driven by a failure in the resolution of inflammation, but also implies a therapeutic anti-atherosclerotic potential of restoring a functional inflammation resolution. The restoration of an endogenous resolution response would have the advantage to obtain the stop signal for the ending of the inflammatory circuits at the right time point. Consequently, the risk for immunosuppression would decrease. The latter notion has received support from experimental studies, in which macrophages exhibit specific lipid mediator release in response to bacterial stimuli [15], and resolvins, in addition to dampening the inflammatory reaction, also enhance bacterial clearance [16].

### Omega-3 PUFAs in atherosclerosis and cardiovascular risk

A possible way to obtain an appropriate resolution of inflammation without immunosuppression would hence be to increase the endogenous production of proresolving mediators. One therapeutic avenue that could lead to this end is to increase the substrate for SPM formation by means of omega-3 supplementation. It has been 40 years since the original observation that the cardioprotective effects of marine diet were largely due to the high omega-3 PUFA content altering lipid mediator metabolism [17]. The discovery of the omega-3-derived SPMs around 20 years later provided another potential molecular mechanism underlying beneficial actions of omega-3 PUFAs [18]. It is however only recently that the link between omega-3, resolvins, and atherosclerosis has been evoked [11, 13, 19–22].

Despite encouraging results from observational studies, randomized controlled trials (RCTs) of different omega-3 PUFA formulations did not consistently show any significant improvement in cardiovascular outcomes [23]. Recently, the REDUCE-IT trial however showed that pure EPA at a higher dose (4 g) compared with previous RCTs provided a 25% risk reduction for cardiovascular events in patients with hypertriglyceridemia of which 71% had prevalent cardiovascular disease [24]. It is in this context interesting that also another omega-3 RCT, coined JELIS, and which reported significant cardiovascular prevention also used EPA albeit at a somewhat lower dose (1.8 g) [25].

Since the REDUCE-IT [24] and JELIS [25] trials point to possible therapeutic potential for EPA, the present review will focus on the EPA-derived SPM resolvin E1 (RvE1) and its effects in atherosclerosis, intimal hyperplasia, and vascular calcification. In particular, targeting the specific RvE1 receptor ChemR23 [26] (also referred to as CMKLR1 and ERV1 [27, 28]) has provided important complementary evidence linking omega-3 PUFA supplementation with EPA to anti-atherosclerotic signalling by means of stimulating the resolution of inflammation.

In addition to the resolution of the immune reaction, it has become increasingly clear that proresolving pathways in atherosclerosis in addition involve direct effects on structural cells of the vascular wall. In particular, recent studies on vascular smooth muscle cells (VSMCs) have reinforced that the resolution of vascular inflammation in atherosclerosis constitutes a close interplay between immune cells and VSMCs.

### Omega-3-metabolism in murine atherosclerosis

Studies in hyperlipidemic mice have supported that dietary omega-3 PUFA supplementation both alter systemic cholesterol levels towards a beneficial lipoprotein profile and are locally incorporated into cardiovascular tissues [29–31]. These findings hence raise the notion that EPA may serve as the substrate to increase the local production of omega-3 PUFA-derived metabolites, which potentially could act locally to resolve atherosclerotic inflammation. Specifically, 12- and 18-HEPE increase after EPA supplementation in apolipoprotein E (apoE)-deficient mice [29, 31], of which 18-HEPE is the precursor for the proresolving mediator RvE1. Importantly, dietary-supplemented EPA appears to preferentially distribute to thin cap atherosclerotic plaques [29], indicating a particular therapeutic potential for plaque stabilization, which will be further discussed below.

### Omega-3-metabolism in human atherosclerosis

The enzymes necessary for metabolizing fatty acids into lipid mediators are locally expressed within human atherosclerotic lesions [32]. Although yet to be established for RvE1, other resolvins are indeed released from human carotid atherosclerotic plaques studied ex vivo [13]. Importantly, the relative abundance of omega-6 and omega-3 PUFAs may determine whether the resulting lipid mediators are either pro-inflammatory, or proresolving [2]. In a study of salivary biomarkers, subjects in which resolvin levels prevailed over leukotriene concentrations had less signs of subclinical atherosclerosis [14]. Furthermore, the increased atherosclerosis associated with variant 5-LO genotypes is completely blunted by a high EPA and DHA dietary intake [33]. Taken together, these results provide some initial indications that findings in murine models of atherosclerosis may translate to human disease.

### RvE1 in atherosclerosis

**Fig. 2:**
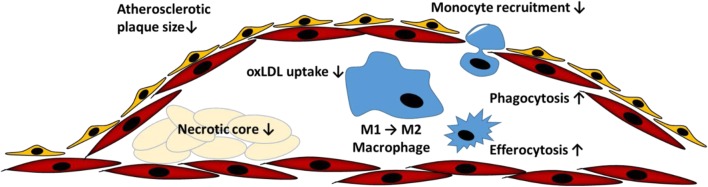
Proresolving effects of resolvin E1 in atherosclerosis

Several of the established proresolving actions of RvE1 may represent a turning point in the atherosclerosis process and may therefore prevent its evolution into chronic inflammation, as depicted in Fig. 2. The potent actions of RvE1-enhanced phagocytosis were first demonstrated in macrophages in vitro and in murine peritonitis models in vivo [34], and have now also been confirmed in macrophages derived from atherosclerotic models [31]. In particular, the phagocytic removal of apoptotic cells, referred to as efferocyotosis, is a hallmark of the resolution of inflammation in the atherosclerotic plaque [35, 36], whereas defective efferocytosis further promotes atherosclerosis and enhances chronic inflammation [37].

Defective efferocytosis is a major driver of necrotic core formation in advanced atherosclerosis [38]. It is therefore of importance that macrophages expressing the ChemR23 receptor for RvE1 are localized in the vicinity of the necrotic core in human atherosclerotic lesions [31], in line with the efferocytotic actions of its ligand [39]. In further support of RvE1 signalling being of importance for the clearance of apoptotic macrophages and necrotic core formation during atherogenesis, transplantation of ChemR23-deficient bone marrow to LDL receptor (LDLR)-deficient mice increases necrotic core size [31].

In contrast to efferocytosis, other macrophage uptake mechanisms exert strong proatherogenic properties, such as phagocytosis of modified lipoproteins [40], platelets [41], and erythrocytes [42]. Most typically in atherosclerosis, uptake of oxidized LDL triggers a strong proinflammatory response in macrophages [40]. It is therefore of note that while RvE1 increases phagocytosis of zymosan, a microbial product, in macrophages from hyperlipidemic atherosclerotic mice, it reduces the uptake of oxidized LDL under the same experimental conditions [31]. Those actions of RvE1 are lost in mice lacking the ChemR23 receptor [31] illustrating that a proresolving ligand may signal through the same receptor to exert opposite effects on different uptake mechanisms.

Since oxLDL uptake and phagocytosis are both of crucial importance for the turning point of the resolution response [2], accumulation and subsequent conversion of EPA to RvE1 in atherosclerotic lesions could hence be anticipated to promote resolution of inflammation. Indeed, administration of RvE1 to animal models has confirmed its anti-atherosclerotic actions in vivo [43, 44]. Similar results have also been reported for DHA-derived proresolving mediators [45].

Double knock-out mice lacking both apoE and the RvE1 receptor ChemR23 exhibit increased atherosclerosis compared with ChemR23 expressing apoE knock-out mice after 8 and 12 weeks of high-fat diet [31]. This is accompanied by an inflammatory plaque phenotype [31], consistent with the RvE1-ChemR23 pathway promoting the resolution of atherosclerotic inflammation. A subsequent study however failed to reproduce these findings under similar conditions using another genetic targeting strategy [46]. It should however be noted that ligand-induced responses were not examined in the latter study and that a disruption of the RvE1 signalling was not confirmed [46]. Nevertheless, these findings illustrate the complexity of proresolving signalling in atherosclerosis, which is regulated by means of both ligand availability, receptor expression, and compensatory mechanisms. In addition to RvE1, ChemR23 is also activated by the adipokine chemerin [47] as well as chemerin-derived peptides [48], which induce differential responses depending on peptide length. Notably, the ChemR23 receptor agonist chemerin-p decreases atherosclerosis after 4 weeks treatment in apoE knock-out mice [49].

### Proresolving signalling in atherosclerotic plaque stability

**Fig. 3:**
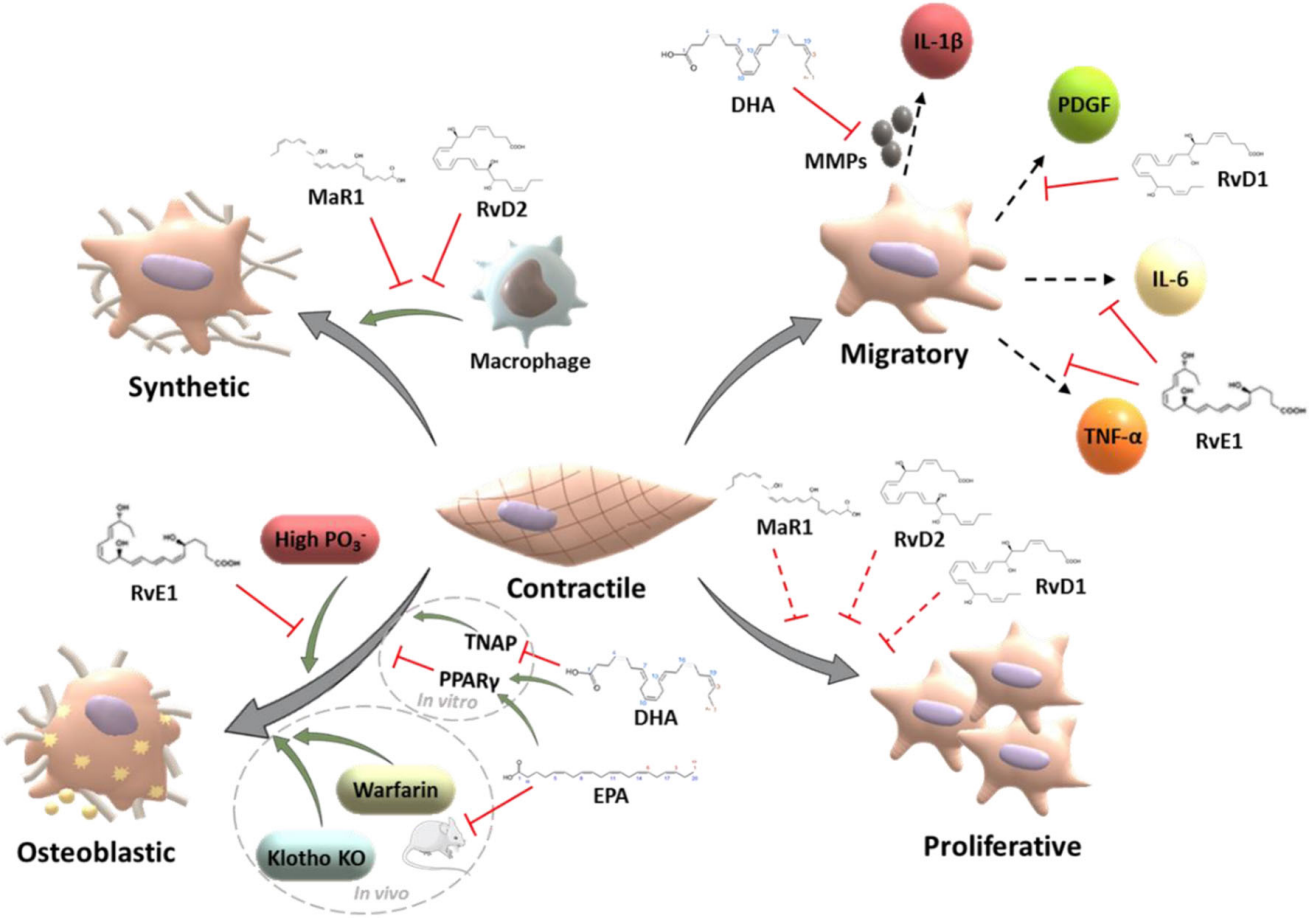
Modulation of vascular smooth muscle cells by proresolving lipid mediators. Some of the pathways underlying EPA- and DHA-induced inhibition of vascular calcification are also depicted (bottom right) Abbreviations: EPA, eicosapentaenoic acid; DHA, docosahexaenoic acid; Rv, resolvin; MaR1, maresin 1

Proresolving mediators do not only serve as regulators of the immune response, but also act on structural cells of the vascular wall to participate in the healing processes as illustrated in Fig. 3. Alterations in the VSMC phenotype, characterized by increased proliferation, migration, aberrant extracellular matrix (ECM) production and degradation, and loss of contractile proteins, are hallmarks of atherosclerosis [50], as well as in the vascular response to percutaneous coronary interventions (PCI) and coronary artery bypass grafting (CABG) [51]. Importantly, the expression of the ChemR23 receptor for RvE1 is not limited to immune cells, but has also been detected in smooth muscle cells in atherosclerotic lesions [31] and in vessels from patients with chronic kidney disease [52].

Changes in VSMC phenotype contribute to the destabilization of the atherosclerotic plaque leading to plaque rupture. As discussed above, the omega-3 PUFA EPA preferentially accumulates in thin cap atherosclerotic lesions [29], indicating a particular potential for its downstream-derived bioactive lipid mediators for plaque stabilization. In addition, the DHA-derived lipid mediators RvD2 and MaR1 increase the number of VSMCs, fibrous cap thickness, and collagen production in murine atherosclerosis, collectively indicating higher atherosclerotic plaque stability [45].

Under homeostasis VSMCs are quiescent and present a low proliferation and migration capacity. However, the inflammatory environment normally observed in the atherosclerotic plaque can promote VSMCs to transdifferentiate towards a migratory and proliferative phenotype [53]. Several studies have shown direct effects of both omega-3 PUFAs and proresolving lipid mediators inhibiting VSMC migration towards a wide array of stimuli including PDGF, TNFα, IL-1β, and IL-6 [54]. Specifically, DHA decreases IL-1β-induced VSMC migration. Mechanistically, this effect of DHA appeared to be mediated by reducing ECM remodelling through the inhibition of elastinolytic MMP activity [55]. RvD1 and RvD2 reduce MMP2 and MMP9 in inflammatory murine abdominal aortic aneurysmal lesions [56]. RvD3 decreases MMPs in neutrophils, supporting that the actions of resolvins in vivo on VSMC migration and ECM remodelling might be partially mediated through the interplay between structural cells and immune cells [57].

Proresolving lipid mediators have direct effects on VSMC migration through different mechanisms. RvD1 inhibits PDGF-induced VSMC migration by inhibiting cytoskeletal rearrangements, which is partially mediated through the receptor ALX/FPR2 [58]. In pulmonary VSMCs, RvE1 reduces TNFα- and IL-6-induced migration and calcium sensitivity [59]. Also, RvE1 inhibits VSMC migration towards PDGF in murine VSMCs in vitro in a dose-dependant manner through its receptor ChemR23, whereas no effect was observed on VSMC proliferation [60]. Interestingly, the lack of effect of RvE1 on proliferation suggests independent pathways in the modulation of VSMC function during resolution. These findings are in line with the modest effects observed with other proresolving lipid mediators in vitro [61, 62], and in sharp contrast to the RvE1-induced effects in animal models of VSMC proliferation in vivo [60]. Taken together, these observations suggest that the inhibition of VSMC proliferation by SPMs may be partially dependent on immune cells, which will be further discussed below. Stimulation of VSMC with macrophage supernatants has indeed supported that disruption of proresolving signalling in macrophages alters VSMC proliferation [63].

In further support of an interplay between VSMCs and immune cells during resolution, RvD2 or MaR1 alters VSMCs collagen production only in the presence of macrophage supernatants [45], suggesting that collagen production was directly affected by omega-3 PUFAs and lipid mediators derived from immune cells. Importantly, this interplay between immune and structural cells appears to be reciprocal, since RvE1 decreases TNFα-induced RANTES production in VSMCs, partially through ChemR23, and thus reducing T cell trafficking [60].

### Vascular calcification

Another hallmark of atherosclerosis is plaque calcification. Calcification results from the nucleation of calcium and phosphate into calcium-phosphate crystals, and is characterized by the phenotypic transformation of VSMCs into osteoblast-like cells [64]. Calcification of coronary atherosclerotic plaques, in the form of spotty micro calcifications, decreases plaque stability, thus increasing the risk of plaque rupture, thrombus formation, and subsequent myocardial infarction [65].

A prospective cohort study has shown that serum levels of omega-3 PUFAs correlate with lower prevalence of coronary artery calcification (CAC) [66]. In addition, a cross-sectional study in 1074 Japanese males with sub-clinical atherosclerosis showed that in those with a CAC score > 300, there was a significant negative correlation between DHA and CAC score [67]. Collectively, those studies suggest a relationship between dietary intake of omega-3 PUFAs and a decrease in atherosclerotic calcification.

EPA and DHA have proven effective at inhibiting calcification in vivo (Fig. 3)*.* Specifically, EPA inhibit warfarin-induced vascular calcification in rats [68] and spontaneous vascular calcification in klotho mutant mice [69]. Mechanistically, some work trying to elucidate the mechanism of action of DHA and EPA has been carried out using calcifying vascular cells (CVCs), a subpopulation of bovine aortic medial cells that undergo spontaneous osteoblast differentiation and calcification. Under these experimental conditions, DHA reduces osteoblastic differentiation, and tissue non-specific alkaline phosphatase (TNAP). DHA promotes the phosphorylation of p38-mitogen-activated protein kinase (MAPK), alongside the activation of the peroxisome proliferator-activated receptor-γ (PPAR-γ) [70]. In line with these results, EPA prevents the β-catenin-induced VSMC trans-differentiation towards osteoblast-like cells through the activation of PPAR- γ [71]. These experiments shed light on the potential pathways involved in the reduction of vascular calcification by omega-3 PUFAs (Fig. 3); however, they do not address whether these actions are mediated directly by DHA and EPA or their downstream-derived proresolving mediators, are receptor dependent, or simply a consequence of changes in the membrane composition.

Recent work from our laboratory has shown that RvE1 inhibits phosphate-induced calcification in VSMCs in vitro through ChemR23, without altering the VSMC phenotype [52]. Importantly, another proresolving ChemR23 ligand, chemerin, was negatively associated with CAC score in CKD patients [72] and, like RvE1, chemerin inhibited phosphate-induced calcification in VSMCs [72].

### Intimal hyperplasia

Although the incidence of intimal hyperplasia after PCI drastically decreased after the introduction of drug-eluting stents, it remains a clinical problem in the pathophysiology of atherosclerosis and restenosis after CABG. Pathologically, intimal hyperplasia comes as an endpoint of the combination of different factors, such as VSMC proliferation and migration, immune cell infiltration into the vessel wall, and aberrant ECM deposition [73]. Hence, targeting proresolution pathways in this context would offer additional advantages in cardiovascular prevention.

**Fig. 4:**
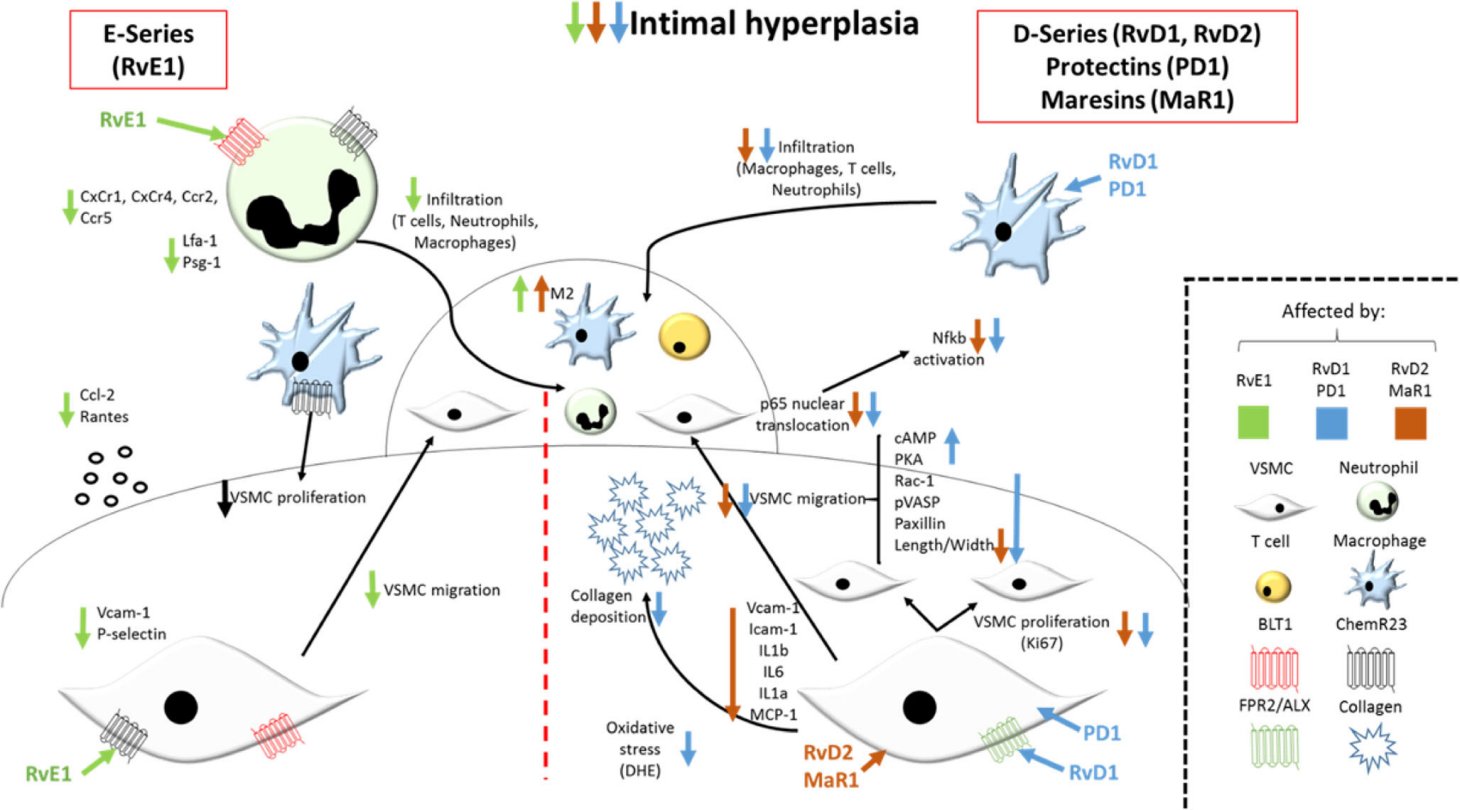
The effects of proresolving lipid mediators on intimal hyperplasia after vascular injury. Abbreviations: CCr, CC chemokine receptor; CxCr, C-x-C chemokine receptor; DHE, dihydroethidium; IL, interleukin; ICAM-1, intercellular adhesion molecule 1; *Lfa-1,* lymphocyte function-associated antigen 1; MaR1, maresin 1; NF-κB, Nuclear factor kappa-B; pVASP, phosphorylated vasodilator-stimulated protein; PD1, protectin 1; Rv, resolvin; VCAM-1, vascular cell adhesion molecule 1

After vascular injury during for example PCI and CABG, leukocytes attach and infiltrate into the vascular wall. Subsequently, macrophages and neutrophils secrete a pool of pro-inflammatory cytokines combined with growth factors that further increase leukocyte recruitment and modulate VSMC behaviour, inducing activation, migration, and proliferation within the site of injury, thus creating an intimal hyperplasia [74]. Omega-3 PUFAs exert beneficial effects to inhibit the development of intimal hyperplasia after vascular injury. In fact, recent studies have demonstrated that those effects of omega-3 PUFAs are largely attributed to their downstream-derived proresolving lipid mediators [54], through pathways mentioned below and depicted in Fig. 4.

#### SPMs reduce intimal hyperplasia by modulating leukocyte behaviour

Recent studies have shown that the actions of SPMs on the development of intimal hyperplasia come as a receptor-specific action involving both leukocytes and structural cells. After femoral artery wire injury in mice, RvE1 specifically signals through BLT1 (and not through ChemR23) in leukocytes, decreasing infiltration of neutrophils, T cells, and macrophages to the site of injury in the vessel wall [72]. RvE1 mediates those effects in leukocytes by downregulating the expression of cytokine receptors as well as decreasing the adhesion molecules to the vascular wall, as illustrated in Fig. 4. On the other hand, ChemR23 on macrophages reduces VSMC proliferation by decreasing the secretion of pro-inflammatory and pro-proliferative cytokines such as TNFα, IL-1β, IL-6, and MMP9 [63]. Furthermore, administration of RvD1 and PD1 after balloon injury in rats [75] or in a vein graft model in rabbits [76], and RvD2 and MaR1 after carotid ligation in mice [62], gave reduced macrophage, T cell, and neutrophil infiltration to the vessel wall after 3–4 days of injury that was accompanied by macrophage polarization to a M2 state (Fig. 4).

#### SPMs reduce intimal hyperplasia by modulating VSMC behaviour

SPMs exert their beneficial effects in intimal hyperplasia by not just signalling on leukocyte but also by directly modulating VSMCs behaviour. For example, after femoral artery wire injury, RvE1 signals through ChemR23 in VSMCs (and not through BLT1 as seen in leukocytes) and reduces VSMC migration, but not proliferation, from the media to the site of injury [60]. This effect has been described to be mediated by the downregulation of VCAM-1 and P-selectin expression as well as a reduction in the pro-inflammatory cytokines CCL-2 and RANTES in VSMC (Fig. 4).

After vascular injury, RvD1 and RvD2 reduce VSMC proliferation as well as migration as a result of increasing levels of cAMP and PKA, and decreasing Rac-1, pVASP, and paxillin [58] as well as reducing collagen deposition [76]. In addition, both RvD1 and RvD2 inhibit p65 nuclear translocation [62, 77], thus reducing NFκB activation and as a consequence, reducing the inflammatory environment by decreasing, for example, IL-1β, IL-6, [61], and CCL-2 expression [62] (Fig. 3).

## Summary and conclusions

The proresolving mediators derived from omega-3 PUFAs may interact at several points of the atherosclerosis process. As discussed above, it is important to take into account both the immune response and the structural cells of the vascular wall when considering the resolution of atherosclerotic inflammation. Specifically, macrophages and VSMCs play a major part in the resolution response, both independently and through close interplay. The beneficial effects of EPA in cardiovascular prevention, together with the potent proresolving and anti-atherosclerotic actions mediated by the EPA-derived lipid mediator RvE1 through its receptor ChemR23, put the spotlight on the potential therapeutic benefits of stimulating the EPA/RvE1/ChemR23 pathway to promote resolution of atherosclerotic inflammation and to improve the outcomes of atherosclerosis-related cardiovascular disease.
